# Report on the Progress of Surgery

**Published:** 1845-12

**Authors:** Henry Ancell


### RECORD OF MEDICAL SCIENCE.

### *Report on the Progress of Surgery.*

BY HENRY ANCELL, ESQ. M. R. C. S. E.

No observer can doubt that Surgery is making rapid progress. While on the one hand new operations are successively discovered for the cure of pathological lesions hitherto deemed irremediable, as the operation for the cure of strabismus, and the subcutaneous section in tenotomy: on the other hand, operations by the knife are superseded by a more rational and scientific, a less objectionable and equally successful treatment of the disease: as in the probable substitution of pressure for incision in the cure of some cases of aneurism. Again, surgical operations which proved unsuccessful and were abandoned in despair in a more infantile period of the art, are now likely to be resorted to successfully in the treatment of incurable maladies, as exemplified in the abdominal section for the extirpation of diseased ovaria. In a more strictly scientific point of view also, surgery is steadily advancing with the progress of physiology and pathology. An improved acquaintance with the phenomena of inflammation especially, derived from microscopical anatomy and micro-chemistry, is leading on to more rational views of treatment. Statistical records are daily furnishing more accurate data of the results of practice, and the innumerable facilities now afforded in all the more civilized communities for extended and multiplied observation are increasing in an incalculable ratio our stock of knowledge. So much is this the case that no individual in active practice can hope to embrace all that is in progress, and all that has been done, even within a limited period, in his own reading and investigation, a circumstance which we feel assured will render the periodical Reports of the Progress of Surgery, which are to form an essential part of this retrospect, acceptable and useful to the surgical practitioner, embracing, as they are intended to do, results—and more especially practical results—in reference to etiology, diagnosis, pathology, and treatment.

1. One of the most recent works that has issued from the press on any general division of the subject of surgery, and by a surgeon, is an interesting volume by Mr. Macilwain, on the Nature and Treatment of Tumours. Mr. Macilwain maintains it to be a pure assumption that cancer, fungus hematodes, and other malignant tumours, are incurable by the powers of nature—and an assumption, moreover, we may add, the tendency of which is the more pernicious as it perverts and discourages the labours of those who are industriously inclined. He takes a threefold view of the causes of tumours:—In the first place they are referable to the food containing something unusual;—secondly, To the assimilating organs acting on the ingesta in some unusual manner;—and lastly, or to both these circumstances combined. His curative intentions are therefore directed to diet, and to a regulation of the various organs which represent the different stages of assimilation. No organ in the body, according to the views of this author, is more frequently in fault than the liver, and this often without any indicative symptoms. A knowledge of this fact is to be arrived at by a careful history of the patient, in order to ascertain what injurious influences the liver has been exposed to at any time, and whether ordinary influences have been so exalted as to become injurious. The influences referred to are especially—sedentary habits, and the free use of alcohol, and of greasy, fatty, and saccharine matter; substances which contain a superabundance of carbon. To regulate disordered liver he tries to diminish the quantity of carbon, except such as is contained in necessary food. A man may eat meat—Mr. Macilwain observes—without fat; butter is unnecessary; so is sugar; and in this way, without depriving the system of anything really necessary to health, the liver may be materially relieved, and this indeed is the great mode of assisting its function. The result of Mr. Macilwain's experience in the treatment of tumours is the smallest possible degree of confidence in local applications, particularly in those which are supposed to excite curative actions in the part, and might be called *positive* remedies. To the judicious management of those which act by excluding injurious agencies, or *negative* remedies, he attaches more importance.

Cancer, so long as the viscera are sound, is a curable disease; and the absorption of malignant tumours generally is under the influence of remedial treatment. Our author gives the case of a lady, aged 39, who consulted him for a tumour of the breast which had been pronounced hopeless. It was a true specimen of carcinoma—very hard, adherent to the subjacent parts, the skin tucked in at the nipple with a dark spot there, slightly abraded, painful, with a sense of drawing; the arm swollen and red in the vicinity of the tumour, and a few drops of blood issuing from the dark spot daily. Her general health was greatly deranged—probably not one function in her whole body performed healthily. The plan of treatment consisted in the use of a very plain diet, the rigid exclusion of sugar and grease of all kinds, friction to the skin, with special avoidance of any interference in the neighbourhood of the tumour. After the cessation of her scanty catamenia, a few leeches were applied to the pubes, and the medicine generally used were aloes and ipecacuanha, with now and then a dose of calomel. The case improved; the tumour became moveable, the suffering from it very trivial, and except when she transgressed in her regimen, although now and then a little uncomfortable, she was generally easy.

The patient relaxing in her obedience, an eminent physician was consulted, who allowed her to "eat what she pleased." The result was, that in a few days she was thrown into a state of the most terrible suffering, for which opium was prescribed without relief; but a return to the former plan was attended with almost immediate amelioration, although she never recovered her former ease. So long as she adhered strictly to the plan, she had almost absolute immunity from pain; but she frequently tampered with impunity. She ultimately died, as the author believes, from her own imprudence, in great agony. Mr. Macilwain gives other cases to illustrate the beneficial effects of regular and daily exercise, a plain diet, and the exclusion of saccharine, oleaginous, and alcoholic matters in malignant tumours. The plan of diet should be "simple, and strictly defined;" and one of his correspondents, Mr. Kingdon, remarks:—I am now convinced that many diseases, which have hitherto been considered incurable, may be cured by that close attention to the minutiæ of function which *does not permit the most insignificant portion of the frame to be overlooked.*"

As respects depositions generally which are not cancerous, Mr. Macilwain now seldom sees any in which he cannot succeed, by strict treatment; sometimes in procuring their absorption, almost always in arresting their progress; and he believes that he has succeeded in promoting the absorption of ten or twelve malignant tumours at least.

The author's observations on the progressive results of the successful treatment of tumours are thus described:—

"The first change observed is, that if there have been much pain, there is a very material and marked diminution, or a total subsidence of it, without the influence of *opium or any other narcotic;* and this, too, when opium and other narcotics have been exhibited in vain. The tumour becomes loosened as to its subjacent connections; and I have more than once seen a tucked-in nipple resume the natural appearance. A change yet more important is, that the tumour which had before, perhaps, presented itself in one mass, with more or less irregularity, becomes broken into portions, so as to feel like separate depositions, intersected as it were by lines of more healthy structure. This is speedily followed by a diminution of the characteristic hardness; so that after a while the carcinomatous character becomes entirely lost. The change progressing, the tumour becomes gradually absorbed, until nothing remains but what, if now examined for the first time, might be taken for an enlarged gland; and this gradually disappearing, the part resumes its ordinary character. Now all this has never happened without a change in some one or more important function to which the treatment has been specially directed, though this has varied in different cases according to the function which, on careful analysis, appeared to be most seriously or primarily affected. If I were called on to name any dietetic measure most general, I should say the diminution of carbon, in the interdiction of grease, sugar, and alcohol. If I were asked what one organ has most frequently appeared to take the lead, I should say the liver."

We have thus given as fair a statement of Mr. Macilwain's views as our space will admit of, because we verily believe that much suffering accrues, if not loss of life, from men holding the most eminent position as surgeons and physicians, distrusting the unquestionable powers of nature, and relinquishing the systematic and scientific treatment of what they deem hopeless diseases. Heterologous formations frequently disappear, to the surprise of the surgeon, without any obvious cause, but not without a cause. These cases establish the justice of our claims for the powers of nature. We believe our readers will regard Mr. Macilwain's views respecting the principles and the details of his treatment not merely as interesting, but as important.

2. A work has also been recently published on the *Treatment of Cancerous Tumours of the Breast without Operation*, in Paris, by S. Tanchou, in which 302 cases of cancer are collected together, as having got well after the employment of various remedies. We find this author boldly maintaining that cancer is not absolutely incurable. He gives, it is true, but three cases of amelioration, and fails to prove his position; yet the cases cited have been admitted by some of his reviewers as demonstrative that it is possible not only to ameliorate cancerous affections, but also to arrest their progress and render them stationary.

3. A work published at Paris by Dr. Jules Guyot in 1842, and reviewed in the *British and Foreign Medical Review* of January last, will doubtless call the more particular attention of surgeons to the important subject of temperature in operations and the treatment of surgical diseases. M. Guyot infers, from numerous experiments, that air, when devoid of any noxious impregnation, has, simply considered, no injurious action on living parts. It may, nevertheless, become injurious in two ways:—1. Mechanically, as by causing the lung to collapse in penetrating wounds of the chest,—by destroying the cellular attachments of the viscera in wounds of the abdomen,—and by distending or compressing organs as in emphysema. 2. As the medium of an injurious temperature, air introduced into the serous and synovial cavities occasions inflammation by its coldness: at the temperature of 36° cent. ($96\frac{4}{5}$) Fahr., the articular, cranial, thoracic, and abdominal cavities, may be opened without danger of inflammation, in so far as the contact of the atmosphere is concerned. By the term "Incubation" as a curative process, M. Guyot implies a temperature of 36° cent. applied to the body by means of heated air. He distinguishes three species:—1. Local or circumscribed incubation adapted to amputations, wounds, &c. 2. Diffuse incubation, acting upon a considerable portion of the body, to re-establish a function, restore the equilibrium of the circulation, &c. 3. General incubation applicable to premature birth, defective development of infants, and various diseases. It may also be continued or intermittent in its application. Various kinds of apparatus are employed for the purpose. This peculiar treatment has been tried and found successful in ulcers, some of which were of an inveterate kind; and in one case of twenty-five years' standing, in a man aged 67, in a large, callous, and fistulous sore, in an immense lacerated wound of the arm. In a case of phlegmonoid erysipelas, terminating in gangrene, two days after the commencement of the treatment, the suppuration was diminished to one half, the wound was covered with granulations, and the fever had ceased. In œdema of the limbs, in phlegmonoid erysipelas, eczema, and a numerous variety of cases, it appears most essentially to influence the cure.

M. Guyot has put his method into practice in *white swelling*, and records cases which seem to indicate that it is worthy of a more extensive trial. A case of chronic rheumatism was cured in twelve days, and a case of periostitis of the tibia in eight days. A malignant or ill-conditioned ulcer of the nose, which had resisted ordinary treatment, was much benefited by incubation; and in another case a cancerous ulcer, of the worst character, also seated on the nose, this treatment modified the sore in a remarkable manner; the mammillated and tuberculated surface contracted its limits, caused the redness and pain to disappear, and predisposed the tissues to cicatrization. The arsenical paste was then applied, and the cure became complete. Thirty-two cases are detailed in which incubation was applied to the stump after various amputations, and the results, as compared with the results of amputation under ordinary treatment in the Parisian Hospitals, were very greatly in favour of the remedy, although there is some reason to doubt whether they were fairly attributable to the artificial temperature.

While on the subject of temperature in the treatment of surgical diseases, we may state that in Mr. Grantham's work, quoted in our extracts, great stress is laid upon the principle of keeping up the normal temperature, or "the vitality" of the superficial structures in inflammation of the ligamentous, tendinous, and cartilaginous parts of the body; and the book contains illustrations of the efficacy of the treatment founded on this principle. Thus, in the after treatment, on reduction of a bad dislocation of the ulna and radius, Mr. Grantham steams and foments the limb for an hour, and then applies a large hot bread-and-water poultice for several days. He observes of this case, which was cured in a few days, "That no blood was lost, nor any cold application used." The treatment was simply keeping up the action of the exhalants and absorbents, so as to remove tumefaction and extravasation without lessening the vital properties pertaining to the low organized textures, as it is the ligamentous and tendinous structures which are injured in all dislocations.

In the operations for removal of ovarian tumours, the surgeon finds it of the first importance to regulate the temperature of the atmosphere. In fact, no one who has witnessed the extent and length of time of the exposure of the abdominal viscera in many of these operations can doubt that their safety has been, to a very considerable extent, secured by attention to this circumstance, and that inattention to it would very decidedly increase their mortality. We have placed this subject thus imperfectly before our readers, convinced that surgeons in this variable and inclement climate are, in the main of their practice, much too negligent on this important point, and in the hope that the future numbers of the "Retrospect" will have to record more favourable results in several departments of surgical practice, when it receives a greater share of attention.

4. Dr. Egan has lately published an elaborate article *On the diagnosis and treatment of syphilitic diseases*, the substance of which he recapitulates as follows:—

"1st. I have observed the simple superficial ulcer, unattended with indurated margin or base, give rise to a papular eruption, pains resembling rheumatism, increased vascularity of the throat, generally accompanied with enlarged tonsils. In this form I have never witnessed the occurrence of rupia, nodes, or ulceration of the back of the pharynx: in this class, which were for the most part treated without mercury, constitutional symptoms occurred far more frequently, but were of a milder description than in those where the opposite plan of treatment was adopted. When topical applicatious fail, mercury is resorted to for the purpose of accomplishing a cure. 2dly. That strong presumptive evidence has been afforded, that the matter of gonorrhœa, in its incipient stage, is capable of producing a mild form of secondary symptoms; but not having been able to substantiate this opinion by the process of inoculation, I cannot, as far as my experience goes, lay it down as an ascertained fact. 3dly. That the excavated ulcer, with indurated margins and base, commonly described as the Hunterian chancre, has, in my limited number of cases, been succeeded by a scaly eruption and excavated ulcers of the tonsils; and that in those cases alone mercury deserves the name of a specific. 4thly. That the phagedenic ulcer, where it has existed *ab initio*, does not owe its characters to any peculiarity of constitution, but to a specific virus, as is evinced in the dissimilarity and inveteracy of the secondary and tertiary symptoms: and that in such cases mercury is decidedly injurious. And, lastly, that all the secondary forms of syphilis, with the exception of iritis, are curable without the aid of mercury; the cure, however, is much more protracted, but relapses far less frequent."

5. There is nothing before us of very great importance in *Thoracic Surgery*. Forty cases of injuries of the chest were the whole number received into Guy's Hospital from January 1, 1843, to December 31, 1844. The series excludes injuries in this region of minor importance treated out of the hospital. Of the 40 cases four terminated fatally, that is to say, 10 per cent. The cases include, simple injuries of the chest; fractures of the ribs, sternum, and clavicle; comminuted fractures; penetrating wounds of the chest; injuries of the lungs; with different complications, as pneumonia, pleuritis, pleuro-pneumonia, emphysema, hemoptysis, collapse, and general obscure inflammatory and other symptoms. The usually admitted principles of treatment were resorted to, as—rest, bandaging, bleeding,—general and local, and purgatives; and to meet special complications and symptoms—calomel and opium, incisions, punctures, brandy and other stimulants, blisters, antimony, diaphoretics, &c. Except that a series of such cases must present novelties in their details, and if faithfully recorded, must be interesting and instructive to the surgical practitioner, this report does not contain anything new in principle, either as respects diagnosis or treatment.

6. The following is an interesting case of injury of the chest, with hernia of the right lung. A child, 13 years old, fell from a considerable height, whilst he was playing, and struck himself against the end of a branch of a tree which had been recently cut. A wound about three inches in length on the anterior part of the right side of the chest was the consequence, extending transversely between the fifth and sixth rib. Dr. Anglo, who was called in immediately, found a considerable quantity of blood from the interior of the chest, and at the same time an elastic tumour about the size of the fist, and of a rosaceous colour, having a transverse wound, and evidently being part of the inferior lobe of the lung. There was much oppression and anxiety, an extreme pallor, the pulse "miserable," the extremities cold, and every thing indicated imminent danger. The protruded portion of the lung was reduced, although with difficulty, and the edges of the wound were brought together with bandages. The patient was three days recovering from the violence of the concussion. Reaction then came on which was met by bleedings and antiphlogistics. The wound soon cicatrized, the pulse recovered its functions, and in six weeks the cure was complete. The French Journals contain several cases in which paracentesis thoracis has been performed with successful results.

7. In *Abdominal Surgery*, Ovariotomy occupies the first place. The revival of the abdominal section for the purpose of removing diseased ovaria, is an important circumstance in the history of modern surgery, and the progress of opinion respecting this important operation, both as to its propriety as a mode of cure, and the various modifications which may be suggested in the manner of performing it, deserve the immediate and most serious attention of every practical surgeon, for, although the general treatment of the disease itself most frequently devolves upon the accoucheur, the formidable nature of the operation will in all cases render it desirable that it should be undertaken by the practical surgeon. We propose to fix the attention of our readers upon the state of our knowledge at the epoch from which this "Retrospect" dates, by referring to Mr. Phillip's paper on the subject.

This gentleman has collected the results of 81 operations:—

| | |
|---|---|
| The tumour was extracted in - - - | 61 cases |
| Adhesions, &c., prevented its extraction in | 15 |
| No tumour was found in - - - - | 5=81 |

Of the whole number:—

| | |
|---|---|
| The recoveries after abdominal section were | 49 |
| The deaths - - - - - - - | 32=81 |

But all the cases in which no tumour was discovered, and the operation was not completed, recovered.

Of the cases in which the tumour was extracted there were:—

| | |
|---|---|
| Recoveries - - - . - - | 35 |
| Deaths - - - - - - | 26=61 |

Of those in which adhesions and other circumstances prevented the removal of the tumour, nine recovered, and six died.

The large incision was resorted to in 55 cases with the following results:—

| | |
|---|---|
| Cured - - - - - - - | 23 |
| Recovered - - - - - - | 6 |
| Died - - - - - - - - | 26=55 |

The small incision in 27, of which there were:—

| | |
|---|---|
| Cured - - - - - - - | 13 |
| Recovered - - - - - - | 7 |
| Died - - - - - - - | 7=27 |

Several years ago, that distinguished physiologist and practitioner, Dr. Blundell, drew up a paper which was read to the Medico-Chirurgical Society, the object of which was to demonstrate what the author then believed to be a fact, that the fears entertained by surgeons were greatly exaggerated respecting the dangers that attend wounds of the peritoneum. We have not seen the paper, but have had opportunities of conversing with its author upon the subject, and we understand that it embraced cases of most extensive laceration of the parietes of the abdomen, with extrusion of the viscera, which had ultimately recovered. The Society declined publishing the paper, but the author has never had reason to alter his own deductions from a consideration of those cases. The recent results of the abdominal section tend very greatly to confirm his views. It is quite true that in many cases of ovarian disease, previous to the period at which an operation is likely to be performed, the whole peritoneum has assumed a pathological condition; but it is equally true that inflammation, and all its consequences, is liable to occur at any period during the progress of ovarian disease, and also, that it is inflammation which is chiefly dreaded by the surgeon, in making extensive incisions through the peritoneum, and in handling and exposing the abdominal viscera. It is not for us to encourage or discourage any particular *methodus medendi*, we are rather the chroniclers of events; and we shall in future collect all the cases which meet the public eye, wherein this operation is resorted to, with a view to place the results before our readers. It may, however, as we conceive, be justly remarked, that according to the evidence before us, the danger of inflammation in these cases, is not so great as we have been taught to believe, and the observation of Dr. Blundell upon this head is perfectly correct.

An additional case is recorded by Dr. Bowles, of Harrison county, Ohio, wherein the incision was nine inches along the linea alba: the omentum was found to be adherent to the tumour, and the latter firmly bound down by adhesions to the bladder and uterus; but all the adhesions were easily separated by the handle of the scalpel. The patient vomited during the operation, producing protrusion of the intestines. To prevent distension of the bowels by flatus, a tube was kept in the rectum. The tumour was solid, and weighed five pounds. No doubt remained of ultimate recovery.

Cases by Mr. Clay, Mr. Lane (unpublished,) and others, since the above record in the *Medico-Chirurgical Transactions*, have come to our knowledge; they are for the most part successful, and will be placed before our readers in our next number.

8. The proposed revival of the treatment of fistula in ano by ligature, according to an improved method, has met with objections. It is asserted to be inapplicable and insufficient in many cases, and also more painful and hazardous than the operation with the knife. Mr. Salmon, after nearly twenty years' experience, denies that the operation by incision is attended by any peculiar hazard from hemorrhage or otherwise. In 248 cases, selected promiscuously, he states that no fatal hemorrhage occurred; in 20 instances only, was there any bleeding which required attention, and in these cases the simplest precaution sufficed to prevent any serious inconvenience. The operation by ligature has, again, been admitted to be applicable in cases of small and single sinuses, but its propriety doubted where large abscesses, with loss of substance, and complicated sinuses tending in different directions, occur. In these latter cases, Mr. Luke admits that ligature offers no better prospect of success than the knife, but, he states, "certainly not less." The probability of success must depend much upon the extent and direction of the communicating sinuses, and upon their complications with ligamentous and osseous structures. In more remediable cases, Mr. Luke remarks, that although, when mismanaged, the ligature may produce protracted pain, yet, when well managed, it neither produces as much pain as the knife, nor as much hazard; particularly where the rectal aperture is barely within reach of the finger. The insertion of the ligature according to the plan recommended, causes scarcely more pain than a common examination; it is at no time necessary to draw it so tight as to cause pain; "during the first few days, or until the slight inflammation of the fistula which succeeds its introduction has attained its maximum amount," it should be left loose; it is then made nearly tense, but not to give pain, and the tension is to be kept at this point by renewed turns of the screw every two or three days. The whole proceeding is to be slowly conducted, and in this consists the essence of the improvement as compared with the mode of applying the ligature in former days. When pain occurs, it usually arises on the accession of inflammation, causing the included parts to swell; the ligature is then to be loosened, which is readily effected by the kind of apparatus employed. We may add, that the operation by the knife is one greatly dreaded in most cases by patients, and frequently deferred in consequence of that dread; and there can be no doubt, that if the surgeon can conscientiously resort, as a general principle, to the treatment by ligature, the subject of this formidable disease will more readily confide in the scientific surgeon, and less frequently fall into the hands of the charlatan.

9. One of the most important surgical subjects which has lately been brought before the consideration of the profession is *the cure of aneurism by pressure.* Several cases of this formidable disease, cured without operation, are quoted in our extracts, and it is most important that the practical surgeon should be made fully aware of the recent results of experience in this method of treatment. Compression effected in various ways has repeatedly been resorted to, for the purpose of arresting or curing aneurism, but we believe that we may state, at once and unequivocally, that the application of pressure upon strictly scientific principles has never had a fair trial. The cases before us justify the opinion that in many cases it will prove an adequate remedy, and will supersede the use of the ligature.

It may be useful to take a cursory view of the objects for which compression has been resorted to in the treatment of aneurism.

Dr. W. Hunter held that when the integuments begin to give way in aneurisms of the aorta, a bandage judiciously applied might preserve life for some considerable time, but looking to the effects of pressure on the arterial tunics, during the gradual dilatation of the aneurismal sac, where it meets with the resistance of the osseous structure, as of the sternum or vertebræ, he came to the conclusion, that in the main, compression or tight bandaging would only aggravate the evil. Dr. D. Munro taught the same doctrine. But in the of false aneurisms, when small, particularly those occurring in the arm from bleeding, Dr. Munro advocates, together with moderate depletion, compression on the part, by means of compresses and bandages, to prevent the blood from flowing into the cyst; the compression to be continued not only till the tumour disappears, but likewise for some time after, lest a relapse should occur; quoting cases which have been cured, but concluding with the remark, that "where such aneurisms are large, and of long standing, this method can have no effect." Guattani and the surgeons of Rome about this period (1750-60,) attached more importance to compression as a remedy, and resorted to it somewhat more systematically in crural and popliteal aneurisms. The latter author recommends gradually compressing the aneurismal tumour, by means of bandages applied more and more tightly from day to day, and gives successful cases. It will be observed that Guattani's method was "direct and energetic" pressure. Subsequently to the Hunterian operation being adopted, the plan of compressing the artery at some distance from the tumour was made trial of by Dubois, Astley Cooper, and others, the object being, by severe and continued pressure, to render the artery impervious. The objections to these modes of applying pressure are stated to be, and no doubt are, positive. It is either insufficient, or it is impossible to protect the neighbouring parts from its influence, and accordingly the flow of blood in the collateral vessels is arrested; it requires to be continued for a great length of time, and is attended with great pain. Hence constitutional disturbance followed by inflammation, ulceration, or sloughing of the compressed parts; in numerous cases the patient has been unable to sustain it, and from other causes it has proved unsuccessful.

We will furnish our readers with a short account of the more scientific application of pressure for the cure of aneurism, lately adopted by Hutton, Cusack, Bellingham, Allen, Greatrex, and Liston, from Professor Miller's *Principles of Surgery.* The pressure is made at the Hunterian site, but is neither constant nor severe. By any suitable apparatus, a moderate degree of pressure is applied to the vessel, at a point where the coats may be expected to be sound, and consequently not prone to ulcerate from various causes.

This is maintained so long as it can be conveniently borne by the patient, but no longer. When uneasy sensations become intense, with swelling and numbness of the limb, and throbbing in the part, the pressure is either slackened or removed. After a time, the parts having recovered, it is re-applied. Again it is removed, and thus the disasters formerly attendant upon the treatment by pressure are avoided. At the same time that the circulation in the aneurism is moderated, the tumour begins to diminish, its pulsation is less, and it feels harder and less compressible; ultimately pulsation wholly disappears, and induration becomes complete, absorption advances, and the cure is obtained with or without a previous state of the vessel. Throughout the treatment absolute repose and decumbency are maintained, with an antiphlogistic regimen. Also, the limb below the compressed point must be uniformly and equally supported by bandaging, lest passive congestion and œdema supervene; and this pressure may, from time to time, be somewhat increased on that part of the limb which includes the aneurismal tumour. The process is one of weeks, not days—gradual, not sudden—interrupted, not continuously progressive. The pressure requires to be neither great nor constant, for we do not desire obliteration even temporary. It is sufficient to moderate, not essential to obstruct the flow of blood. The treatment is conducted rather as if itself were not the agent of cure, but only the means whereby the spontaneous cure may be originated and favoured.

The objections still advanced by Professor Miller to this improved method of applying pressure are:—The protracted period and ultimate uncertainty of the cure. If improperly conducted, it is in every point of view inferior to the ligature. It is less capable of general application, since many systems cannot tolerate pressure; our experience of its use is in its infancy.

The last objection of Professor Miller is exactly that which will render it incumbent on us, in the future numbers of the "Retrospect" to place before our readers an abstract of all the cases, whether successful or unsuccessful, that may for some time to come meet the the public eye. This is the only way by which profit can be made to accrue to the great body of surgeons by the experience of individuals. At present it will suffice to say that in 1831 Assalini published a case of popliteal aneurism cured by pressure. In November last, Dr. Hutton, of Dublin, recorded seven cases treated by pressure, one only of which was unsuccessful. Giraldès in his memoir cites fifteen cases, including those just referred to, of aneurism treated successfully by compression of the femoral artery, and he remarks that the mean duration of the treatment was 24 days, the minimum 5 days, and the maximum 90 days; and this author judiciously suggests that one circumstance which ought to plead strongly in favor of compression, and recommend it to the attention of surgeons, is the contingencies which await the operation by ligature. The ligature is not an infallible remedy; too frequently the disease returns after the operation, of which circumstance instances are cited upon the authority of Cooper, Brodie, Roux, and Lenoir; and serious accidents, as consecutive hemorrhage, wounds, and inflammation of the veins, gangrene, and death, have been the consequence. Such being the fact as relates to the treatment now resorted to by surgeons, the new plan ought at all events to be tried for the present, in every case, before resorting to the operation.

The advantages of compression over ligature are, that it is exempt from all danger; it is, so far as the evidence enables us to judge, more universally successful; it is applicable to certain cases of aneurism to which the ligature is not, and to some in which the operation by ligature would be likely to be followed by unfavorable results, as in the case of very large aneurisms which compress the collateral circulation. It is applicable in cases wherein the ligature is contraindicated from disease of the arterial system, and in cases of the "aneurismal diathesis;" also in cases of spontaneous aneurism in broken-down constitutions, in which the surgeon would perform an operation only with the greatest reluctance; and lastly, its employment, if ever it should happen to be unsuccessful, does not preclude the subsequent operation by ligature.

We conclude this article with a quotation from Dr. Bellingham's explanation of the mode of applying the pressure.

"Instead of employing a single instrument, we employ two or three, if necessary; those are placed upon the artery leading to the aneurismal sac, and when the pressure of one becomes painful it is relaxed, the other having been previously tightened; and by thus altering the pressure, we can keep up continued compression for any length of time." . . .

The instrument "consists of an arc of steel covered with leather, at one extremity of which is an oblong, padded splint; the other extremity terminates in a nut, containing a quick screw, to which a pad, similar to that of the tourniquet, is attached. . . . The principle is extremely simple; so much so, that the patient can regulate its application himself, and it can be made of every size, so as to compress any vessel within the reach of compression."

10. The subject of tracheotomy and the removal of foreign bodies from the larynx excited great interest at the period when Mr. Brunel's case was before the public. In that remarkable case the patient suggested the idea of inverting the body so as to give the same facility to the passage of the substance out of the tube which it had to get in, but the experiment demonstrated that the human system is not a mere machine—a vital spasmodic action closed the glottis and rendered the experiment unsuccessful, until the art of the surgeon was resorted to for the performance of tracheotomy. A case, however, has been recorded in the *Northern Journal of Medicine* from which it appears that the idea of the mechanician will sometimes succeed, and that the knife of the surgeon may be superseded. A shilling slipped through the glottis of an individual, giving rise to comparatively little inconvenience. It seemed to the patient to be fixed at the cricoid cartilage, and that it would return if he were placed on his head:—

"The man was placed with his shoulders against the raised end of a pretty high sofa, and then being seized by three of the most powerful of those present by the loins and thighs, he was rapidly inverted, so as to bring the head into the dependent position, and after a shake or two, Dr. Simpson at the same time moving the larynx rapidly from side to side, the shilling passed into the mouth and fell upon the floor." The relief was instantaneous and complete.

In our own immediate neighbourhood a piece of cotton wool passed into the trachea of a lady from a carious tooth; a medical man was called in, and precipitately ran out for another surgeon to perform tracheotomy. In the interval, the husband believing that his wife was at the point of death, inverted her body, the foreign substance was expelled, and before the return of the doctors she was perfectly restored. It should not be forgotten, however, that the foreign substance in falling from the trachea might fix itself in the glottis and cause almost instant death, so that the inversion of the body cannot be considered a safe measure unless the surgeon is at hand with his scalpel.

11. The various foreign and domestic journals during the last six months contain the usual amount of information in the shape of lectures, essays, discussions, cases, &c.; but we believe we have extracted the most important novelties. There are some interesting instances of those cases which involve what are usually styled the minor operations of surgery, which are by no means undeserving attention. The following is an instance in point:—A lady from the country had a carious tooth filed to admit a pivot to an artificial tooth. This trifling operation, which was attended with little pain, was followed by an abscess in the jaw, for which the extraction of the root was recommended; but as it was a superior canine tooth, with a deep root, and as the stump presented no hold, the most skilful dentists would not undertake the extraction. According to the advice of M. Recamier, M. Maisonneuve made an incision in the mucous membrane; and after exposing the maxillary bone, he divided the bone along the alveola. This operation is infinitely less painful than those resorted to by dentists with their various instruments. The root being thus exposed, M. Maisonneuve laid hold of it with a hook, and it was removed with great ease.

12. Operations, novel in character, or remarkable for their boldness, are sometimes resorted to *once* even by judicious surgeons, and are justifiable, although judging from results we may arrive at the conclusion they ought never to be repeated. These and the more rash experiments of the ignorant or reckless, like rare and extraordinary cases, frequently throw light upon the phenomena of nature, or serve as beacons for the future. Instances of one or more of these different classes of surgical cases will be found in that part of our volumes set apart for "extracts," and we may add to those contained in our present volume two cases of "Puncture of the intestines to relieve the agony of distension in supposed internal strangulation," lately recorded by Sir George Lefevre.

The lady of an officer of high rank had been suffering for some time with disordered digestion, when she was suddenly seized with violent vomiting and purging, and fecal matter was discharged by mouth. To this succeeded constipation and tympanitis; the latter being so distressing that it was resolved to puncture the bowels. Large quantities of gas escaped: the patient felt immediately relieved from her extreme sufferings. She died the same day or the following."

"A lady, the mother of six children, was in the family way of the seventh, and in about the fourth or fifth month of utero-gestation. She had for years been in the habit of neglecting her bowels, and retaining her fæces for five or six days, and even longer with impunity. She complained of sudden pains in the bowels, which she took to be colicky, and used some domestic medicine. The pains increasing, and the bowels continuing locked, blood was taken from the arm, and leeches applied very freely to the part. No relief was afforded. The abdomen became very tense, and the pains returned at repeated intervals. She expressed herself thus, that she had borne six children, and that the united pains of all her labours were not so excruciating as any one of the pains under which she suffered. All means had failed in procuring her relief. Six or seven medical men were in constant attendance upon her. Injections, warm baths, cold effusions, bleeding, opium in large doses, nothing relieved her.

"It was, therefore, upon the idea only of shortening her sufferings, that it was proposed to puncture the intestines. A trocar was thrust into the colon, some gas escaped, and she exclaimed, 'I can breathe now.' Fæces soon filled up the canula; the spasms returned, but in rather diminished force. She sank in about fourteen hours after the operation, suffering to the last.—*Half-Yearly Abstract of the Medical Sciences.*

